# Early multimodal neuromonitoring is associated with outcomes in critically ill patients with hemorrhagic stroke

**DOI:** 10.3389/fneur.2026.1845374

**Published:** 2026-05-21

**Authors:** Ana Ferreira, Joana Magalhães, Pedro Alberto Silva, Tiago Gregório, Elisabete Monteiro, Marek Czosnyka, Celeste Dias

**Affiliations:** 1 Department of Neurosurgery, ULS São João, São João Local Health Unit, Porto, Portugal; 2 RISE-Health, Department of Clinical Neurosciences and Mental Health, Faculty of Medicine, University of Porto, Porto, Portugal; 3 Department of Internal Medicine, ULS São João, São João Local Health Unit, Porto, Portugal; 4 Stroke Unit, ULS Gaia e Espinho, Gaia and Espinho Local Health Unit, Vila Nova de Gaia, Portugal; 5 Department of Intensive Care Medicine, ULS São João, São João Local Health Unit, Porto, Portugal; 6 Department of Clinical Neurosciences, University of Cambridge, Cambridge, United Kingdom

**Keywords:** cerebral autoregulation, cerebral perfusion pressure, critical care, intracerebral hemorrhage, neuromonitoring

## Abstract

**Introduction:**

Early outcome prediction in critically ill patients with spontaneous intracerebral haemorrhage (ICH) remains challenging. Invasive multimodal neuromonitoring may offer prognostic insights.

**Methods:**

We conducted a single-centre retrospective observational study including critically ill ICH patients admitted to the Neurocritical Care Unit between January 2015 and September 2021. Neuromonitoring variables were analysed during the first 48 h and included intracranial pressure (ICP), cerebral perfusion pressure (CPP), pressure reactivity index (PRx), and optimal CPP (CPPopt). Six-month functional outcomes were assessed using the modified Rankin Scale (mRS) score and dichotomized as severe if death or vegetative state (mRS score 5–6) or nonsevere (mRS score 1–4).

**Results:**

Among 65 patients with a mean age of 62.6 ± 13.2 years, the mean PRx was significantly higher in patients with a severe outcome (0.27 ± 0.27 vs. 0.12 ± 0.18; *p* = 0.008). The difference between real CPP and CPPopt was negative in the severe outcome group and positive in the non-severe group (−4.79 ± 13.6 mmHg vs. +3.99 ± 15.3 mmHg; *p* = 0.020). The ICP values did not differ significantly between groups and were <21 mmHg in both groups. In multivariable analysis, higher PRx (OR 1.05, 95% CI 1.01–1.10; *p* = 0.025) and negative CPP–CPPopt deviation (OR 0.95, 95% CI 0.91–0.99; *p* = 0.028) were independently associated with severe outcome.

**Discussion:**

Although ICP is the most extensively studied neuromonitoring parameter, it may not be the most informative parameter associated with outcome in this population during the early phase. Critically ill patients with ICH who exhibit impaired cerebrovascular reactivity (PRx > 0.2) or lower CPP than CPPopt within the first 48 h were associated with a higher likelihood of poor functional outcomes at 6 months. These findings support the hypothesis that advanced neuromonitoring parameters may provide additional prognostic information beyond mean ICP values in this population.

**Clinical trial registration:**

ClinicalTrials.gov, identifier NCT05501613.

## 1 Introduction

Spontaneous intracerebral haemorrhage (ICH) is a devastating neurological condition associated with high mortality and long-term disability. Critically ill ICH patients admitted to neurocritical care units often require mechanical ventilation and invasive brain monitoring due to their neurological severity and systemic instability. Early identification of patients at higher risk of poor outcomes remains challenging, and prognostic markers tailored to this population are limited (3, 6, 11, 23).

Invasive neuromonitoring has become a cornerstone for managing most forms of acute brain injury, including ICH (11, 16). Traditionally, intracranial pressure (ICP) thresholds have guided clinical decisions, and current guidelines from the American Heart Association / American Stroke Association (AHA/ASA, 2022) and the European Stroke Organization (ESO, 2023) recommend maintaining cerebral perfusion pressure (CPP) between 60–70 mmHg to prevent secondary injury. However, increasing evidence suggests that generalized ICP or CPP targets may fail to address individual cerebral pathophysiology (11, 25).

Studies have proposed that cerebral autoregulation status—evaluated with pressure reactivity index (PRx) and used to determine optimal CPP (CPPopt)—may offer more personalized insight into cerebral haemodynamics (4, 8, 9, 27). These parameters allow clinicians to adjust perfusion goals according to each patient’s autoregulatory responses. Moreover, available data suggest that patient-specific variations in ICP, rather than absolute values, may better reflect the brain’s compensatory reserve and prognostic potential, particularly in ICH, where the contribution of ICP to outcome appears less linear than in traumatic brain injury (30).

Despite a growing interest in individualized neuromonitoring strategies, data concerning the prognostic relevance of PRx, CPPopt, and ICP variability in the early phase of critical ICH management remain limited. Our present study aimed to evaluate the relationship between early multimodal neuromonitoring variables, particularly during the first 48 h after ICU admission, and long-term functional outcomes in critically ill patients with spontaneous ICH.

## 2 Materials and methods

### 2.1 Study design and settings

This single-centre, retrospective observational study included neuromonitoring signal recordings and clinical data from patients with spontaneous intracerebral haemorrhage (ICH) admitted to the Neurocritical Care Unit (NCCU) between January 2015 and September 2021. The study is reported according to the STROBE guidelines. The protocol was registered on ClinicalTrials.gov (NCT05501613) and approved by the institutional Ethics Committee (no. 286/19). The requirement for written informed consent was waived in accordance with national regulations for retrospective analyses of anonymised data.

### 2.2 Participants

Patients were eligible if they presented with primary or aggravated ICH associated with neurological deterioration (Glasgow Coma Scale ≤8) requiring endotracheal intubation and intraparenchymal ICP monitoring. In our institution, the decision to initiate invasive neuromonitoring is based on clinical severity, including decreased level of consciousness (typically GCS ≤ 8), need for mechanical ventilation, and suspected risk of intracranial hypertension or impaired autoregulation. The exclusion criteria included secondary causes of haemorrhage, known pregnancy, less than 24 h of valid neuromonitoring recordings, or missing clinical data.

All patients were admitted to the NCCU under sedation and mechanical ventilation, underwent intraparenchymal ICP probe placement (Codman®), the standard practice in our institution for ICP monitoring, and were managed with continuous multimodal brain monitoring. Clinical protocols in this unit include target-CPPopt management guided by autoregulation assessment using PRx, along with analgosedation, normoventilation, and strict blood pressure control.

**Figure 1:**
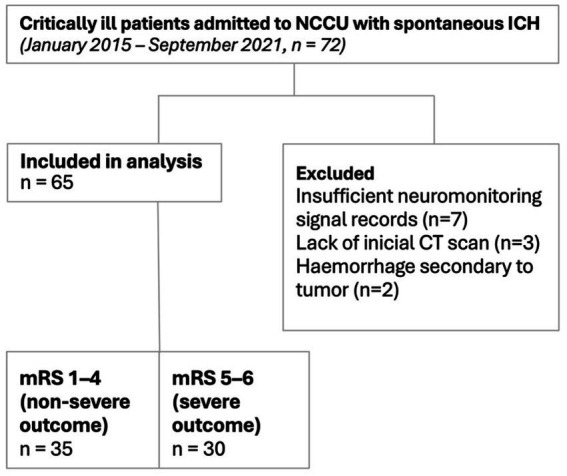
Diagram of patient selection and outcome groups. Flowchart illustrating patient inclusion, exclusion criteria, and division into outcome groups according to the modified Rankin Scale (mRS) score at 6 months.

All eligible patients were consecutively included during the study period, reducing selection bias. Neuromonitoring data were acquired using standardized hardware and a single integration platform (ICM+), ensuring consistency in signal processing. In addition, all patients analysed were managed within the same neurocritical care unit under uniform clinical protocols. To minimize information bias, only patients with valid neuromonitoring recordings and complete clinical data were included in the final analysis (Figure 1).

### 2.3 Data acquisition and monitoring

Data from the first 48 h of multimodal neuromonitoring were collected using ICM + software (Cambridge Enterprise, Cambridge, UK ®), as the integration data system to record and store complete monitoring signals.

The variables of interest were ICP, CPP, PRx, CPPopt, and percentage of time (%time) over the critical value of ICP (21 mmHg), over the PRx (0.2) and under or over CPPopt (−5 and +5 mmHg) and the dose over the critical value for ICP, PRx and CPPopt. Specifically, PRx was calculated as the moving Pearson correlation coefficient between slow waves of ICP and arterial blood pressure, with positive values over 0.2 reflecting impaired cerebrovascular pressure reactivity. CPPopt was derived using the ICM + algorithm as the CPP value associated with the lowest PRx within a given monitoring window, representing the estimated individualized CPP range at which autoregulation is best preserved. “Dose” metrics were calculated as the cumulative burden above or below predefined thresholds, integrating both the magnitude and duration of deviation from the threshold, conceptually corresponding to the area under the curve over time (7).

Demographic and clinical data included age, sex, mRS score before admission, as well as data concerning previous comorbidities, such as high blood pressure, neurologic, lung, hepatic, renal or cardiovascular diseases, smoking, alcohol abuse, and use of antiplatelet and/or anticoagulant medication. Clinical status at admission was documented using the Glasgow Coma Scale (GCS) and Simplified Acute Physiology Score II (SAPS II) (15, 20) scores as measures of the overall severity of the acute disease. The primary haemorrhagic lesion’s location and volume, as well as the option for neurosurgical procedures such as craniotomy/craniectomy or external ventricular drainage (EVD), were also documented. The ICH Score, frequently used for risk-stratification of 30-day mortality for ICH patients, was calculated for all patients and used as a clinical grading scale (12, 17). The length of stay (LOS) at the NCCU was also documented.

### 2.4 Study size

The study sample size reflects an opportunistic cohort based on the number of eligible patients admitted during the study period. A preliminary pilot analysis using institutional data suggested that approximately 28 patients would be sufficient to detect clinically meaningful differences in neuromonitoring parameters with 80% statistical power (10). The final cohort exceeded this estimate, including 65 patients with complete neuromonitoring and outcome data.

### 2.5 Outcome assessment

Functional outcome at 6 months was assessed using the modified Rankin Scale (mRS) and was dichotomized as mRS 5–6 (severe outcome: severe disability or death) versus mRS 1–4 (non-severe outcome). This prespecified dichotomy was chosen to reflect the clinical reality of critically il ICH patients, in whom the likelihood of achieving meaningful recovery is limited (24, 28, 29).

### 2.6 Statistical analysis

Continuous variables were compared using independent-samples t-tests when normally distributed and when the assumption of homogeneity of variance was met. These data are presented as the mean and standard deviation (SD). The non-parametric Mann–Whitney U test was used for data not normally distributed or for time-related variables, and results are reported as median and interquartile range (IQR). Categorical variables are described as absolute values and relative frequencies and were compared by univariable analysis using the chi-square test or Fisher’s exact test. A multivariate binomial logistic regression model was used to assess the association between early neuromonitoring variables and functional outcomes at 6 months while controlling for potential confounding variables. To minimise the risk of overparameterization given the sample size, the number of variables included in the multivariable model was restricted, considering both statistical significance in univariable analyses and clinical relevance. Statistical significance was defined as *p* < 0.05. Data processing and analysis were performed using ICM + software and R statistical software (version 4.2.1).

## 3 Results

A total of 72 patients were screened, and 65 were included in the final analysis after the exclusion criteria were applied. Patient selection and outcome groups are shown in Figure 1.

The mean age was 63 ± 13.2 years, and 63.1% were male. Most patients presented with deep supratentorial haematomas (66%), with a mean haematoma volume of 38.0 ± 27.9 cm^3^ and a median Glasgow Coma Scale (GCS) score of 7 [IQR 6–8] at admission. Intraventricular haemorrhage (IVH) was present in 63.1% of patients. Surgical haematoma evacuation and/or decompressive craniectomy were performed in 44.6% of patients, and external ventricular drainage (EVD) was used in 53.8% due to hydrocephalus. A total of 18.5% of patients had no neurosurgical procedures.

The median length of NCCU stay was 28 days for survivors. Among those who died in the NCCU, the median time to death was 18 days. The overall NCCU mortality was 17%, and the mortality rate at 6 months reached 32.3%. Tracheostomy was performed in 50.8% of patients.

At the 6-month follow-up, 30 patients (46.2%) had a severe outcome (modified Rankin Scale [mRS] score 5 or 6, highly dependent or deceased, respectively), and 35 patients (53.8%) had an mRS score between 1 and 4 (Table 1).

**Table 1:** Descriptive statistics of demographic and clinical data of critically ill ICH patients.

Patient data	n (%), mean (SD) or median [IQR]
n	65
Age	62.6 ± 13.24
Male	41 (63.1)
Female	24 (36.9)
Previous functional status (Modified Rankin Scale)	
mRankin 0	39 (60)
mRankin 1	21 (32.3)
mRankin 2	4 (6.2)
mRankin 3	1 (1.5)
Previous comorbidities	
High blood pressure	48 (75.4)
Current smoker	16 (24.6)
Alcohol abuse	26 (40)
Neurological disease	27 (41.5)
Lung disease	10 (15.4)
Hepatic disease	6 (9.2)
Renal disease	6 (9.2)
Cardiac disease	29 (44.6)
Anticoagulants	12 (18.5)
Antiplatelets	22 (33.9)
Clinical features at admission	
GCS Score at hospital admission: 3–8	24 (36.9)
9–12	21 (32.3)
13–15	20 (30.8)
SAPS II score	43.1 (11.88)
ICH	
Location	
Supratentorial	57 (87.7)
Lobar	11 (16.9)
Lenticulocapsular	23 (35.4)
Thalamic	20 (30.8)
Pure Intraventricular	2 (3.1)
Infratentorial	8 (12.3)
Volume (mL)	
Supratentorial	38.0 ± 27.9
Infratentorial	16.3 [14.77]
IVH	41 (63.1)
Craniotomy/Craniectomy	29 (44.6)
EVD	35 (53.8)
No neurosurgical procedure	12 (18.5)
Outcome	
LOS NCCU (days)	
Alive, discharge from NCCU (*n* = 54)	28 [13.3]
Deceased at NCCU (*n* = 11)	18 [8]
Tracheostomy	33 (50.8)
Modified rankin scale score at 6 months	
mRankin 1	1 (1.5)
mRankin 2	2 (3.1)
mRankin 3	13 (20)
mRankin 4	19 (29.2)
mRankin 5	9 (13.8)
mRankin 6 (deceased)	21 (32.3)

Data are presented as the *n* (%), mean (SD) or median [IQR]. GSC, Glasgow Coma Scale, ICH, intracerebral haemorrhage; IVH, intraventricular haemorrhage; LOS NCCU, length of stay at the neurocritical care unit; mRS, modified Rankin scale; SAPS II, simplified acute physiology score.

There were no differences between outcome groups regarding age, sex, and previous comorbidities, except for a history of renal disease, which was present only in the severe outcome group. Also, there were no differences concerning ICH location, ICH volume, IVH, clinical presentation, SAPS II scores, EVD and surgical procedure (Table 2).

**Table 2:** Comparison of demographic, clinical features for 6-month functional outcome.

	n (%), mean ± SD or median [IQR]		
Demographic and clinica variables	mRS score 1–4	mRS score 5–6	*p*-value
n	35 (53.8)	30 (46.2)	
Age (y)	61.5 ± 13.76	63.8 ± 13.86	0.496
Male	21 (51.2)	20 (48.8)	0.579
Female	14 (58.3)	10 (41.7)	
Previous comorbidities			
High Blood Pressure	27 (55.1)	22 (44.9)	0.936
Current Smoker	13 (59.1)	9 (40.9)	0.667
Alcohol abuse	15 (60.0)	10 (40.0)	0.554
Anticoagulation	7 (58.3)	5 (41.7)	0.730
Anti-aggregation	9 (40.9)	13 (59.1)	0.093
Neurologic disease	12 (48.0)	13 (52.0)	0.344
Lung disease	3 (30.0)	7 (70.0)	0.079
Hepatic disease	4 (66.7)	2 (33.3)	0.560
Renal disease	0 (0)	6 (100)	0.004*
Cardiac disease	14 (50)	14 (50)	0.447
Clinical features at admission			
First GCS score at admission			0.127
3–8	9 (37.5)	15 (62.5)	
9–12	13 (61.9)	8 (38.1)	
13–15	13 (65)	7 (35)	
SAPS II score	41.2 ± 11.40	45.3 ± 12.25	0.088
ICH location			0.554
Supratentorial	31 (54.4)	26 (45.6)	
Lobar	7 (63.6)	4 (36.4)	
Lenticulocapsular	13 (56.5)	10 (43.5)	
Thalamic	10 (47.6)	11 (52.4)	
Pure intraventricular	2 (100)	0 (0.0)	
Infratentorial	4 (50.0)	4 (50.0)	
ICH volume			
Supratentorial	38.4 ± 28.67	37.5 ± 27.62	0.451
Infratentorial	15.3[38.42]	18,3[18.64]	0.271
IVH	20 (48.8)	21 (51.2)	0.284
Craniotomy/Craniectomy	16 (55.2)	13 (44.8)	0.847
EVD	16 (45.7)	19 (54.3)	0.155

Data are presented as the *n* (%), mean ± SD or median [IQR]. *Statistically significant p value considering *p* < 0.05. EVD, external ventricular drain; GCS, Glasgow Coma Scale; ICH, intracerebral haemorrhage; IVH, intraventricular haemorrhage; mRS, modified Rankin scale; SAPS II, simplified acute physiology score.

The mean ICP and the real CPP were not significantly different between the non-severe outcome and the severe outcome groups (ICP: 9.3 ± 4.33 vs. 10.6 ± 7.01 mmHg, *p* = 0.371; CPP: 89.3 [7.94] vs. 85.2 [8.14] mmHg, *p* = 0.075). In both groups, the mean ICP was lower than 21 mmHg. The ICP-related variables, i.e., the ICP maximum value, %time of ICP > 21 mmHg, and ICP dose, did not differ between the groups. Patients with severe outcome exhibited significantly greater PRx values (0.12 ± 0.18 vs. 0.27 ± 0.27, *p* = 0.008), spent more time with a PRx > 0.2 (40.5 ± 23.40% vs. 54.9 ± 25.91%, *p* = 0.023), and had a lower real CPP relative to the CPPopt (+3.99 ± 15.3 mmHg vs. −4.79 ± 13.6, *p* = 0.020). The time spent with the CPP below the CPPopt was also significantly greater in the severe outcome group (25.5 [44.32]% vs. 59.7 [63.84]%, *p* = 0.022).

In both outcome groups, CPP and CPPopt values were significantly higher than the guideline-recommended CPP reference range of 60–70 mmHg (*p* < 0.001).

**Figure 2:**
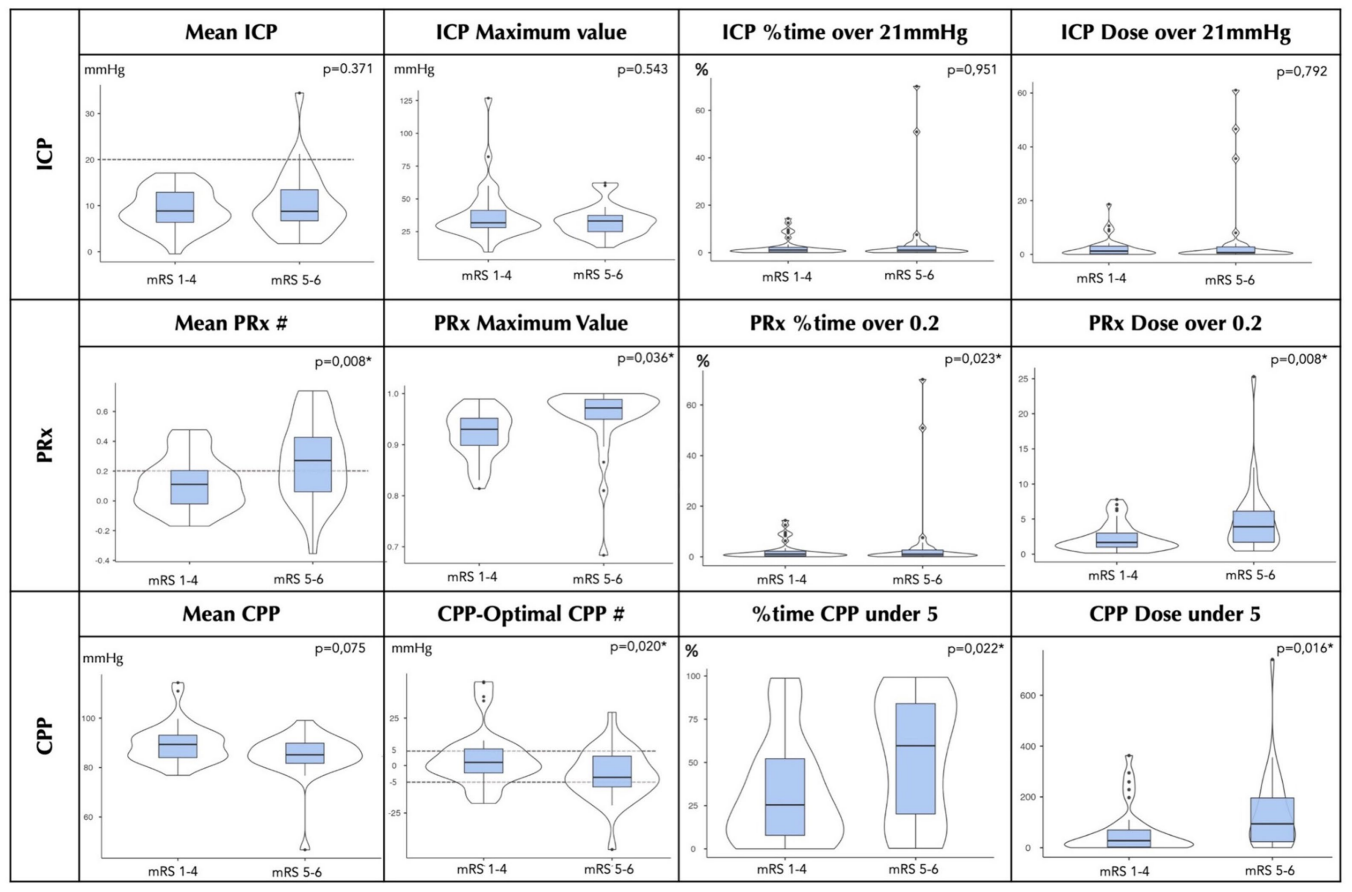
Box and violin-plot graphics of the first 48 h of neuromonitoring distribution for ICH patients by functional status at 6-months: mRS score 1–4 and mRS score 5–6. The variables considered for multimodal analysis are shown in the #. *Statistically significant *p* value. CPP, cerebral perfusion pressure; ICP, intracranial pressure; mRS, modified Rankin scale; PRx, pressure reactivity index; % time, percentage of time.

The neuromonitoring data collected during the first 48 h are summarized in Table 3 and illustrated in Figure 2, which presents box and violin plots of their distribution during this period.

**Table 3:** Initial 48-h neuromonitoring association with 6-months functional outcome.

	*n* (%), mean±SD or median [IQR]		
48 h signal record	mRS score 1–4 35 (53.8)	mRS score 5–6 30 (46.2)	*p* value
ICP Mean	9.3 ± 4.33	10.6 ± 7.01	0.371
ICP Max value	31.8 [13.2]	33.2 [12.5]	0.543
ICP % time >21	1.09 [1.92]	1.03 [2.18]	0.951
ICP dose >21	1.24 [2.83]	0.723 [2.41]	0.792
PRx Mean	0.12 ± 0.178	0.27 ± 0.272	0.008*
PRx max value	0.920 ± 0.046	0.951 ± 0.068	0.036*
PRx % time >0.2	40.5 ± 23.40	54.9 ± 25.91	0.023*
PRx dose >0.2	1.76 [2.00]	3.90 [4.42]	0.008*
CPP Mean	89.3 [7.94]	85.2 [8.14]	0.075
CPPopt Mean	89.4 [13.9]	92.0 [17.4]	0.253
CPP-CPPopt	3.99 ± 15.3	−4.79 ± 13.6	0.020*
% time CPP-CPPopt <5 mmHg	25.5 [44.32]	59.7 [63.84]	0.022*
% time CPP-CPPopt >5 mmHg	27.5 [52.71]	14.5 [34.90]	0.034*
% time without CPPopt	71.7 ± 18.9	76.8 ± 16.8	0.268
Dose CPP-CPPopt <5 mmHg	27.9 [67.01]	94.3 [172.0]	0.016*
Dose CPP-CPPopt >5 mmHg	8,84 [50.5]	0.306 [17.4]	0.069
Dose without CPPopt	215 [348]	387 [388]	0.112

Data are presented as the *n* (%), mean±SD or median [IQR] if they are normally or non-normally distributed, respectively. *Statistically significant p value. The cutoff was *p* < 0,05. CPP, cerebral perfusion pressure; CPPopt, optimal cerebral perfusion pressure; ICP, intracranial pressure; ICP Max, intracranial pressure maximum; PRx, pressure reactivity index; % time, percentage of time.

In multivariable analysis, higher PRx (OR 1.05, 95% CI 1.01–1.10; *p* = 0.025) and negative CPP–CPPopt deviation (OR 0.95, 95% CI 0.91–0.99; *p* = 0.028) were independently associated with severe outcome. The mean PRx and CPP-CPPopt were selected as representative of each statistically significant group of neuromonitoring parameters (Table 4).

**Table 4:** Multivariable logistic regression analysis for 6-month severe outcome.

Model variables	OR	CI 95%	*p* value
Renal disease	1.00	0.997–1.004	0.726
Mean PRx	1.05	1.01–1.10	0.025
CPP-CPPopt	0.95	0.91–0.99	0.028

CI 95, 95% confidence interval; CPP, cerebral perfusion pressure; CPPopt, optimal cerebral perfusion pressure; PRx, pressure reactivity index. The table shows the odds ratio (OR), 95% CI and *p*-value for each predictor. The significant predictors are indicated by *p* < 0.05.

The predicted 30-day mortality according to the ICH Score was compared with the observed 30-day and 6-month mortality in this cohort. For patients with an ICH Score ≥2, the observed 30-day mortality was substantially lower than predicted, as shown in Table 5.

**Table 5:** Mortality by ICH score, 30-day and 6-month of the present cohort.

ICH score	Expected mortality at 30-days by ICH score	n (%)	Mortality 30-days	Mortality 6-months
Total		65/100%	11 (17%)	21 (32%)
0	0%	2/3.1%	1 (50%)	1 (50%)
1	13%	15/23.1%	3 (20%)	4 (26.7%)
2	26%	24/36.9%	2 (8.3%)	5 (20.8%)
3	72%	22/33.8%	5 (22.7%)	10 (45.5%)
4	97%	2/3.1%	0 (0%)	1 (50%)

Data presented as *n* (%). ICH score, intracerebral hemorrhage score.

## 4 Discussion

The American Heart Association recommends postponing early prognostic judgments after intracerebral haemorrhage (ICH) and advocating for sustained optimal care during the acute phase to improve outcome prediction accuracy (11). Under this context, early cerebral physiological data may provide valuable prognostic information, especially in critically ill patients.

In this study, neuromonitoring parameters linked with impaired cerebrovascular autoregulation and negative deviations from optimal cerebral perfusion pressure, when observed during the first 48 h of multimodal neuromonitoring were significantly associated with poor functional outcomes at 6 months. These associations support the hypothesis that early physiological signals may contribute to improved prognostic stratification.

A higher mean PRx, greater time spent with a PRx > 0.2, and a higher dose of impaired autoregulation were significantly more prevalent in patients with severe disability or death (mRS 5–6). Although CPP differed from CPPopt for more than 70% of the monitoring time, greater negative deviations from CPPopt (CPP − CPPopt < −5 mmHg), as well as a higher cumulative dose and longer duration below this threshold, were associated with worse outcomes. Conversely, time spent with the CPP above CPPopt was more frequent in the non-severe outcome group.

These findings are consistent with analogous data from traumatic brain injury and subarachnoid haemorrhage (SAH) populations, where deviation from the CPPopt, especially in the negative direction, has been associated with worse outcomes and lower brain tissue oxygenation (2, 18). Our study contributes to this body of work by reinforcing the relevance of these parameters in a spontaneous ICH cohort. Importantly, the absolute values of ICP and CPP were not independently associated with 6-month outcomes. The ICP remained consistently below the critical threshold of 21 mmHg, and the CPP levels exceeded the guideline-based target of 60–70 mmHg in both outcome groups. These findings likely reflect the standardized ICP management and PRx-guided CPP optimization applied in our unit.

Some authors have reported that a nonspecifically higher CPP could be related to better outcomes for ICH patients (22, 26). However, the risk of a “rightward” shift in the autoregulatory curve for patients who chronically experience high blood pressure may increases the risk of cerebral hypoperfusion if blood pressure / CPP management is not taken into account (14, 22). In addition, although some authors have questioned the utility of ICP monitoring in ICH patients with preserved ICP levels as a whole, our findings support its continuous use (5, 19, 21). Even in the absence of critical ICP elevations, the data derived from ICP sensors, including PRx and CPPopt or ICP derived compliance information offer dynamic insight into cerebrovascular status and perfusion adequacy (31).

Given the heterogeneity of autoregulatory capacity in this group of patients, often with comorbid chronic hypertension, our results suggest that a universal CPP target may be insufficient. Instead, individualized CPP goals based on real-time autoregulatory status may represent a potential approach for a more precise and protective haemodynamic management. Given the retrospective design, causal relationships cannot be inferred. These findings generate hypotheses for future prospective studies and clinical trials evaluating CPPopt-guided strategies in ICH.

The traditional clinical score, the ICH Score, did not accurately predict 30-day or 6-month outcomes. This score, which is designed for broader populations, underperformed in this critically ill cohort, a finding the authors acknowledged as a possibility (12). For patients with an ICH Score ≥2, the observed 30-day mortality was substantially lower than predicted, suggesting that the model may overestimate early mortality risk in patients receiving aggressive, protocolized care. This is reinforced by the fact that the present cohort included truly severe clinical cases, as the long-term functional dependence remained high (50.8% of patients requiring at least temporary tracheostomy and only 4.6% achieving good recovery,mRS 1–2, at 6 months).

Taken together, these findings support the integration of early neuromonitoring data into future prognostic models.

### 4.1 Limitations

Although all patients were managed in a specialized neurocritical care unit under standardized protocols, the retrospective and single-centre design may limit generalizability to other settings and less severe cases.

Ongoing prospective validation studies (ClinicalTrials.gov ID: NCT05771662) are underway to address these concerns.

The restriction of neuromonitoring data to the first 48 h may have limited the detection of plausible delayed secondary insults or dynamic autoregulatory changes over longer time intervals, but was used to address a particularly relevant timeframe and increase coherence in the subgroup comparison.

PRx analysis under altered cranial compliance is debated. Thirty-five patients (53,85%) had an EVD and three patients (4,62%) underwent decompressive craniectomy in the first 48 h. Although PAx may be preferable in this specific setting, it was not recorded systematically (1, 13). To preserve consistency across the cohort, PRx was retained as the primary autoregulatory index for all patients.

Additionally, this cohort does not account for patients excluded due to early withdrawal of care decisions at the time of presentation and/or admission at the emergency department. These patients may represent a more severe clinical subgroup with potentially worse outcomes.

Our outcome dichotomization strategy (mRS 1–4 vs. 5–6) diverges from the more conventional approach (mRS 0–3 vs. 4–6). This decision was intentional and aimed to reflect the clinical profile of critically ill ICH patients, in whom meaningful recovery to independence is relatively uncommon. In this context, grouping mRS 3–4 with non-severe outcomes may better capture the spectrum of survivable states that are often limited by the extent of the primary injury, while mRS 5–6 represents clearly devastating outcomes. Nevertheless, this choice may limit comparability with other studies and is acknowledged as a limitation.

## 5 Conclusion

In this retrospective study of critically ill patients with spontaneous ICH, early impaired cerebrovascular autoregulation (PRx > 0.2) and negative deviations from the CPPopt during the first 48 h of multimodal neuromonitoring were significantly associated with poor 6-month functional outcomes. In contrast, absolute ICP and CPP values alone were not predictive. These findings suggest that autoregulatory indices such as the PRx and the gap between the measured CPP and individualized CPPopt may be considered early prognostic markers in this high-risk population.

## Data Availability

The raw data supporting the conclusions of this article will be made available by the authors, without undue reservation.
